# Two Compound Heterozygous Variants in *SNX14* Cause Stereotypies and Dystonia in Autosomal Recessive Spinocerebellar Ataxia 20

**DOI:** 10.3389/fgene.2020.01038

**Published:** 2020-09-24

**Authors:** Nuno Maia, Gabriela Soares, Cecília Silva, Isabel Marques, Bárbara Rodrigues, Rosário Santos, Manuel Melo-Pires, Arjan PM de Brouwer, Teresa Temudo, Paula Jorge

**Affiliations:** ^1^Unidade de Genética Molecular, Centro de Genética Médica Jacinto de Magalhães (CGM), Centro Hospitalar Universitário do Porto (CHUP), Porto, Portugal; ^2^Unidade Multidisciplinar de Investigação Biomédica (UMIB), Instituto de Ciências Biomédicas Abel Salazar (ICBAS), Universidade do Porto, Porto, Portugal; ^3^Unidade de Genética Médica, Centro de Genética Médica Jacinto de Magalhães (CGM), Centro Hospitalar Universitário do Porto (CHUP), Porto, Portugal; ^4^Serviço de Neuropatologia, Centro Hospitalar Universitário do Porto (CHUP), Porto, Portugal; ^5^Department of Human Genetics, Donders Institute for Brain, Cognition and Behaviour, Radboud University Nijmegen, Nijmegen, Netherlands; ^6^Serviço de Neurologia Pediátrica, Centro Hospitalar Universitário do Porto (CHUP), Porto, Portugal

**Keywords:** intellectual disability, Autosomal Recessive Spinocerebellar Ataxia 20, *SNX14* gene, cerebellar hypoplasia, exome sequencing, complex genomic rearrangement

## Abstract

Autosomal Recessive Spinocerebellar Ataxia 20, SCAR20, is a rare condition characterized by intellectual disability, lack of speech, ataxia, coarse facies and macrocephaly, caused by *SNX14* variants. While all cases described are due to homozygous variants that generally result in loss of protein, so far there are no other cases of reported compound heterozygous variants. Here we describe the first non-consanguineous SCAR20 family, the second Portuguese, with two siblings presenting similar clinical features caused by compound heterozygous *SNX14* variants: NM_001350532.1:c.1195C>T, p.(Arg399^*^) combined with a novel complex genomic rearrangement. Quantitative PCR (Q-PCR), long-range PCR and sequencing was used to elucidate the region and mechanisms involved in the latter: two deletions, an inversion and an AG insertion: NM_001350532.1:c.[612+3028_698-2759del;698-2758_698-516inv;698-515_1171+1366delinsAG]. *In silico* analyses of these variants are in agreement with causality, enabling a genotype-phenotype correlation in both patients. Clinical phenotype includes dystonia and stereotypies never associated with SCAR20. Overall, this study allowed to extend the knowledge of the phenotypic and mutational spectrum of SCAR20, and to validate the role of Sorting nexin-14 in a well-defined neurodevelopmental syndrome, which can lead to cognitive impairment. We also highlight the value of an accurate clinical evaluation and deep phenotyping to disclose the molecular defect underlying highly heterogeneous condition such as intellectual disability.

## Introduction

Autosomal Recessive Spinocerebellar Ataxia 20 (SCAR20, OMIM #616354) was first described in a Portuguese consanguineous family as a disorder characterized by severe ataxia, intellectual disability (ID), lack of speech, coarse facies, macrocephaly, skeletal abnormalities and cerebellar hypotrophy (Sousa et al., 2014). In the same year, Thomas and co-authors identified the Sorting Nexin-14 gene (*SNX14*), described seven affected patients belonging to two distinct families, including those previously reported by Sousa et al. (2014), and widened the SCAR20 phenotypic spectrum describing seizures and deafness (Thomas et al., 2014). To date, 19 distinct homozygous pathogenic *SNX14* variants have been identified, including point mutations and deletions, affecting at least 47 individuals from 25 unrelated consanguineous families (Thomas et al., 2014; Akizu et al., 2015; Jazayeri et al., 2015; Karaca et al., 2015; Shukla et al., 2017; Trujillano et al., 2017; Al-Hashmi et al., 2018; Bryant et al., 2018).

Herein, we describe the first non-consanguineous family with two siblings affected by SCAR20, carrying two *SNX14* compound heterozygous variants, of which only one, p.(Arg399^*^), was identified by exome sequencing. Phenotypic similarity to SCAR20, prompted further characterization of the other allele that subsequently was found to contain a complex genomic rearrangement in *SNX14*: NM_001350532.1:c.[612+3028_698-2759del;698-2758_698-516inv;698-515_1171+1366delinsAG]. Interestingly, siblings also show dystonia and stereotypies, besides the pathognomonic SCAR20 clinical features, representing the first implication of *SNX14* in movement impairment. Further functional studies are warranted to understand the underlying molecular mechanisms.

## Materials and Methods

### Clinical Report

**FIGURE 1:**
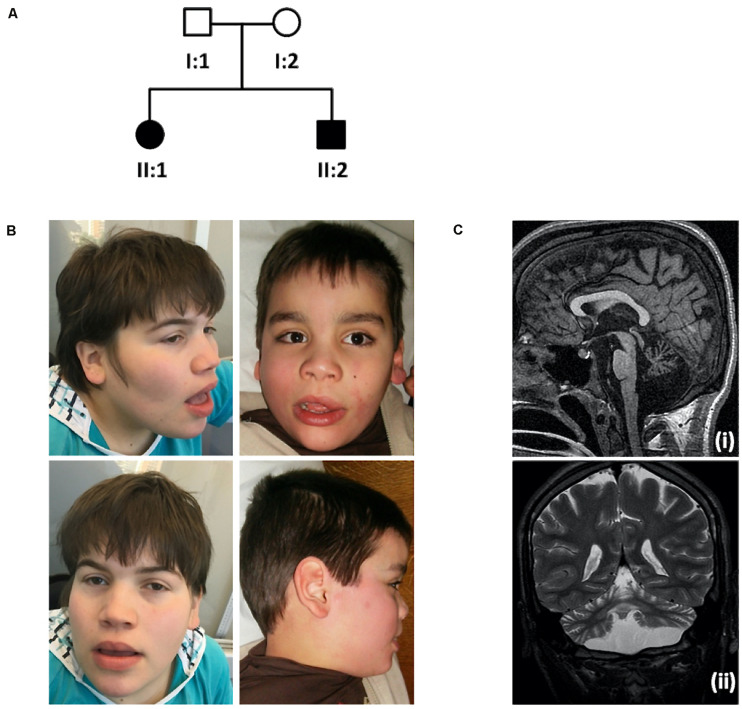
Family pedigree, pictures showing craniofacial features and brain MRI scan. **(A)** Family pedigree. **(B)** Photographs of the index case (II:1, left), at 18-year-old, showing macrocephaly, high forehead, hypertelorism, depressed nasal bridge, large base to nose, anteverted nares and thick lips and younger brother (II:2, right), at 8-year-old, presenting with similar phenotypic features. **(C)** Brain MRI scan from patient II:2: Sagittal T1 SPGR (i) and coronal T2-weighted (ii) images showing diffuse cerebellar atrophy, involving both the vermis and the hemispheres, as well as pontine atrophy. There is no evidence of dentate calcification. Brain MRI form patient II:1 showed overlapping abnormalities.

Patient II:1 is the first female child of a healthy non-consanguineous Portuguese couple (Figure 1A). She is currently 29-year-old. She was referred to a pediatric neurology consultation at 2 months for febrile seizures. Family history is negative for neurodevelopmental disorders except for a paternal second-cousin with Prader-Willi syndrome. Gestation and delivery were uneventful except for gestational diabetes at 37 weeks, born at 39 weeks of gestation with weight, 4000 g, length, 51 cm and occipitofrontal circumference (OFC), 36.5 cm. At 9 months she was very hypotonic with no head control; Growth parameters were normal except for OFC. Additional features include diminished reflexes in the lower limbs, bilateral talipes equinovarus with a dystonic hallux, kyphosis, neurosensorial hearing loss, macrocephaly, and facial dysmorphisms: high forehead, hypertelorism, depressed nasal bridge, large base to nose, anteverted nares and thick lips (Figure 1B). She never acquired independent walking or speech and severe intellectual disability was evident. Although speechless she communicates with the eyes and hands. She also had frequent dystonic manual stereotypies with hands apart.

The male patient II:2, currently 19-year-old, was referred for examination at 3 months when hypotonia and lack of head control were noted. Gestation and delivery were uneventful, born at 40 weeks of gestation with weight, 3250 g; length, 50 cm and OFC, 36.5 cm. Psychomotor development was also severely delayed, with no independent walking or speech acquisition. Macrocephaly with normal additional growth parameters, talipes, diminished reflexes in the lower limbs, bilateral talipes equinovarus with a dystonic hallux, dystonic posture hands, kyphosis, hand stereotypies and facial features similar to his sister were also present (Figure 1B). He was also very sociable and communicated with facial expressions.

Brain MRI (Figure 1C) showed diffuse cerebellar atrophy, involving both the vermis and the hemispheres, as well as pontine atrophy, normal spectroscopy and absence of other malformations. Previous normal investigations included metabolic studies such as lysosomal storage parameters, karyotype, array comparative genomic hybridization (aCGH). Following medical genetics referral, the family was selected for research based on the observed putative autosomal recessive inheritance pattern.

### Molecular Investigation

Genomic DNA (gDNA) was obtained from peripheral blood of index, brother and parents, using salting out methods (Miller et al., 1988).

#### Exome Sequencing and Copy Number Variants (CNVs) Calling

Exome sequencing (ES) was performed in the index case (II:1). Exome libraries were captured using SureSelect V5-post Kit (Agilent Technologies, Santa Clara, CA, United States) and 100 bp paired end sequencing was performed using the Illumina HiSeq 2000/2500 (Illumina, San Diego, CA, United States). Genome Analysis Toolkit (GATK v3.4.0) was used to assemble raw data of FASTQ file format onto the University of California Santa Cruz (UCSC) Genome Browser^1^ - human assembly: February 2009 (hg19 - NCBI build GRCh37) and variant alleles were annotated using SnpEff (SnpEff_v4.1 g) (Cingolani et al., 2012).

Copy number variants calling based on ES data was performed using CoNIFER^2^ (Krumm et al., 2012). CNVs with an absolute *Z*-score greater than 1.7 were considered for analysis. All deletions were considered disruptive, as were duplications in known fully penetrant microdeletion/duplication regions and intragenic CNV duplications. CNVs that overlapped known regions of partial penetrance were considered separately (Firth et al., 2009).

#### Variant Filtering, *in silico* Analyses and Segregation Studies

Variants passing following filters were selected for clinical correlation: (a) frequency < 1% (dbSNP, GnomAD Browser, and local databases); (b) gene component, that is, exon and canonical splice acceptor or donor sites; (c) non-synonymous consequence; (d) *in silico* deleteriousness and spliceogenic effect predictions, using tools: (i) combined Annotation Dependent Depletion scoring^3^ (CADD threshold ≥ 15) (Kircher et al., 2014); (ii) SpliceSiteFinder-like (SSF, normal score threshold ≥ 70 for SDS and SAS) (Shapiro and Senapathy, 1987); (iii) MaxEntScan (MES, normal score threshold ≥ 0 for SDS and SAS) (Yeo and Burge, 2004); (iv) NNSPLICE (NNS, normal score threshold ≥ 0.4 for SDS and SAS) (Reese et al., 1997); and (v) GeneSplicer (GS, normal score threshold ≥ 0 for SDS and SAS) (Pertea et al., 2001). Variants nomenclature follows the Human Genome Variation Society (HGVS) recommendations^4^ (den Dunnen and Antonarakis, 2000).

Segregation studies of the putative pathogenic variants identified by ES were performed by Sanger sequencing analysis using the BigDye^®^ Terminator v3.1 cycle sequencing kit (Applied Biosystems^TM^, Foster City, CA, United States), after purification of the PCR products with Illustra^TM^ ExoStar^TM^ 1-Step, (GE Healthcare Life Sciences^®^, Little Chalfont, United Kingdom).

#### Screening for Low Covered Regions, Genomic Quantitative PCR (Q-PCR) and Long-Range PCR

Integrative Genomics Viewer (IGV) software, version 2.2.11 (Robinson et al., 2011) was used to manually analyze exome reads coverage-depth. Low covered exons were then sequenced for detection of SNVs or indels, and a low cycle number PCR was performed to screen for short CNVs. Symmetric and asymmetric amplification studies were preformed essentially as described above.

Comparative C^*T*^ method (ΔΔC^*T*^) was used to detect constitutional copy number variants in *SNX14* gene. The *TTR* exon 2 was used as endogenous control. Q-PCR assay was carried out using the PowerUp^TM^ SYBR^®^ Green Master Mix (Thermo Fisher Scientific, Waltham, MA, United States) and 7500 fast Real-Time PCR System (Applied Biosystems^TM^), following manufacturer’s instructions.

To ensure equivalent efficiency of amplification between each pair of primers, the average threshold cycle value (C^*T*^) was calculated based on the C^*T*^ obtained for each of the two replicates from a log_10_ dilution series of gDNA (20, 10, 5, 2, and 1.25 ng/μl). Data obtained were subjected to linear regression analysis through the semi-logarithmic regression and calculation of the linear correlation value (*R*^2^). Only primers that lead to *R*^2^ values close to 1 were considered.

In the final computational analysis of the relative copy number variants of each exon, the average C^*T*^ and associated standard deviations (SD) was calculated for each sample. These values were normalized by the endogenous control, finding the ΔC^*T*^ and respective SD values. The values of ΔΔC^*T*^ (and respective SD) were determined by the difference between the ΔC^*T*^ of all samples and the normal control. The relative quantification (RQ) was finally determined by the formula 2^–ΔΔ*C*^*T*^^ (or 2^–ΔΔ*C*^*T*^^ ± SD), considering RQ = 1 for the normal control (Weksberg et al., 2005).

To amplify the genomic region encompassing *SNX14* introns 7 to 13, by long-range PCR, the RANGER DNA Polymerase (Bioline reagents, London, United Kingdom) was used, following the manufacturer’s instructions.

#### Transcript Analysis

RNA was obtained from peripheral blood samples from both patients, using 5 PRIME PerfectPure^TM^ RNA Blood Kit (Fisher Scientific International, Inc., Hampton, NH, United States). A region harboring exons 5 to 15 was amplified using SuperScript^®^ One-Step - RT-PCR with Platinum^®^ *Taq* kit (Invitrogen^TM^, Carlsbad, CA, United States) following manufacturer’s instructions. PCR products were separated on a 2% agarose gel, purified using Corning^®^ Costar^®^ Spin-X^®^ plastic centrifuge tube filters (Merck KGaA, Darmstadt, Germany), and further sequenced as described above.

## Results

**FIGURE 2:**
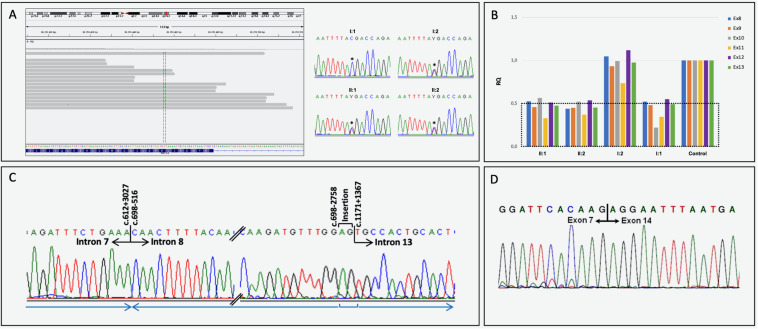
Molecular results. **(A)** Heterozygous nonsense *SNX14* variant NM_001350532.1:c.1195C>T, p.(Arg399*) visualization using IGV, and electropherograms of partial exon 14 sequence generated by Sanger sequencing, with the identified heterozygous variant (*) in the index case (II:1), brother (II:2) and mother (I:2). A normal genotype was identified in the father’s SNX14 exon 14 (I:1). **(B)** Quantitative analysis (Q-PCR) showing a decrease in exons 8 and 13 copy number in individuals II:1, II:2 and I:1. **(C)** Partial electropherograms of the genomic region neighboring the rearrangement breakpoints and respective numbering (indicated above corresponding nucleotide): NM_001350532.1:c.[612+3028_698-2759del;698-2758_698-516inv;698-515_1171+1366delinsAG]. The blue arrows indicate the orientation of each genomic fragment. **(D)** SNX14 transcript analysis performed using lymphocyte-derived RNA of the index case. The wild-type RT-PCR amplicon was amplified in the mother and several controls, while a second smaller amplicon (less 599 bp) was yielded in the patients and father. Sequence trace of the smaller PCR product corresponding to exon 7 - exon 14 junction in the paternally inherited allele, with the absence of exons 8 to 13 (r.613_1171del). This frameshift variant introduces a new stop codon at position 47: p.(Val205Argfs*47);

**FIGURE 3:**
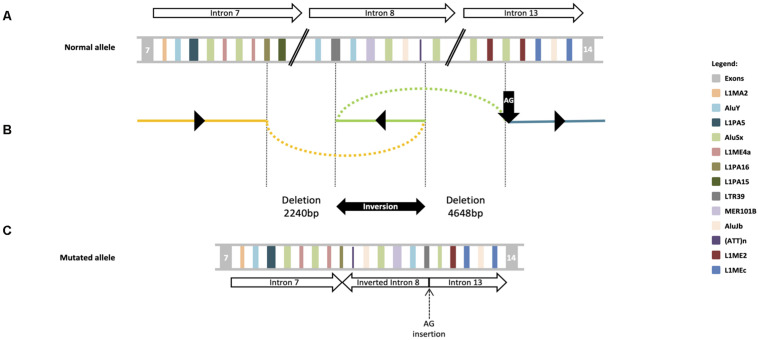
DEL-INV-DELINS structures at the *SNX14* locus. **(A)** Schematic diagram of exons flanking the rearrangement breakpoints; the statement the partial intron 8 inversion, is followed by the 4648 bp deletion, occurred between introns 8 and 13. Repetitive retroelements are labeled by a unique color. Upper arrows indicate *SNX14* introns and respective orientation. **(B)** Schematic depiction of the genomic genomic complex rearrangement: c.[612+3028_698-2759del;698-2758_698-516inv; 698-515_1171+1366delinsAG]. The 2240 bp deletion occurred between introns 7 (yellow line) and 8 (green line), with breakpoints at L1PA16 and LTR39 elements; the partial intron 8 inversion, is followed by a 4648 bp deletion, that occurred between introns 8 and 13 (blue line), within a AluSx and a AG insertion (vertical black arrow). Black triangles indicate the orientation of each fragment at the mutated allele. **(C)** A potential structure of the mutant allele is shown in the lower panel in relation to the canonical genomic structure at the top. Lower arrows indicate each *SNX14* introns and respective orientation.

Based on clinical phenotype patients were diagnosed with intellectual disability-coarse facies-macrocephaly-cerebellar hypotrophy syndrome. After ES data filtering, the previously published *SNX14* nonsense variant NM_001350532.1: c.1195C>T, p.(Arg399^*^), described as pathogenic (Akizu et al., 2015; Shukla et al., 2017; Bryant et al., 2018) (ClinVar: RCV000170506.3) was identified. Segregation studies confirmed that this variant was maternally inherited (Figure 2A). These reports as well as the notable clinical phenotype, prompted further *SNX14* investigations. However, CNV screening and low-covered regions analyses revealed absence of pathogenic variants. Yet, a low-cycle number (*n* = 21) amplification of *SNX14* exons 8 and 13 suggested a reduced amplification yield in DNA samples from both patients and father when compared to mother’s DNA. A Q-PCR assay was performed, confirming a low copy number amplification of *SNX14* exons 8 to 13 shared by the two siblings and father’s samples when compared with control and mother’s samples (Figure 2B). Long-range PCR allowed the characterization of the genomic region breakpoints, revealing a complex rearrangement that includes two deletions, an inversion and an AG insertion, described as NM_001350532.1:c.[612+3028_698-2759del;698-2758_698-516inv;698-515_1171+1366delinsAG] (Figure 2C). Expression analysis enabled the identification of a shorter transcript with a deletion encompassing *SNX14* exons 8 to 13: r.613_1171del, p.(Val205Argfs^*^47) (Figure 2D). Repetitive retroelements neighboring the breakpoints were identified using RepeatMasker (Figure 3; Smith et al., 2015). However, the origin of this complex rearrangement remains to be elucidated, as the well-known mechanisms underlying such phenomena, i.e., non-homologous end joining (NHEJ), homologous recombination (HR), polymerase theta-mediated end joining (TMEJ), microhomology-mediated end joining (MMEJ), fork stalling and template switching (FoSTeS) and microhomology-mediated break induced replication (MMBIR), cannot explain *per se* this occurrence (Wang et al., 2006; Lee et al., 2007; Lieber, 2008; McVey and Lee, 2008; Hastings et al., 2009; Hustedt and Durocher, 2016; Schimmel et al., 2019).

## Discussion

We report the first non-consanguineous SCAR20 family with two affected patients, carrying compound heterozygous pathogenic variants. ES allowed the identification of a known heterozygous pathogenic variant NM_001350532.1: c.1195C>T, p.(Arg399^*^), while a second heterozygous pathogenic variant, NM_001350532.1:c.[612+3028_698-2759del;698- 2758_698-516inv;698-515_1171+1366delinsAG], r.613_1171del, p.(Val205Argfs^*^47), was characterized following a long range PCR.

Complex genomic rearrangements, such as the described herein, are believed to be caused by errors in the DNA double-stand breaks (DSBs) repair system (Carvalho and Lupski, 2016). NHEJ, HR and the recently discovered TMEJ are some of the DSBs repair mechanism already known (Wang et al., 2006; Lieber, 2008; Hustedt and Durocher, 2016; Schimmel et al., 2019). Alu-Alu recombinations are especially common (Sen et al., 2006; Han et al., 2008), which lead us to speculate that an Alu pairing can be the inciting event. This however, cannot explain the full *SNX14* rearrangement, as the deletion breakpoints are within divergent types of retroelements, LTRs (one of the oldest primate-specific L1 sequences) and Alu sequences precluding juxtaposition of homologous domains. Since a LINE-1 element appears partially deleted, it is plausible to hypothesize that a SINE-VNTR-Alu (SVA) might be in the origin of this mutated allele, as SVAs are presumably mobilized by *trans* LINE-1 reverse transcriptase (Hancks and Kazazian, 2010). However, without a stretch of a new retrotransposon or a polyA tail to embody a newly retrotransposed sequence, this also seems unlikely. Interestingly, pathogenic CNVs in other neurodevelopmental genes such as *PLP1, MECP2*, *APP*, and *SNCA*, are believed to be caused by defects in microhomology-mediated DNA repair mechanism (Lee et al., 2007). In fact, Lee et al. (2007) characterized a genomic rearrangement in *PLP1* gene, originated by FoSTeS, a mechanism previously known to cause inversions accompanied by duplications and deletions in other organisms such as *Escherichia coli* (Lee et al., 2007). Although we cannot explain the exact origin of the rearrangement, probably caused by a cascade of errors, it is tempting to speculate that *SNX14* gene is prone to the emergence of complex rearrangements as it harbors a large number and variety of repetitive retroelements.

The loss of non-coding RNAs (ncRNAs) at the *SNX14* locus could contribute toward the severity of the SCAR20 phenotype, as ncRNAs are implicated in the regulation of protein coding host gene expression (Boivin et al., 2018). However, according to the NONCODE database^5^ (assessed at June 22nd 2020) (Liu et al., 2005) there is no ncRNA at these positions in *SNX14* gene, so their involvement is unlikely.

Notwithstanding recent developments in sequencing technologies, this kind of genomic rearrangements are overlooked and encumber the identification of the genetic defect particularly in highly heterogeneous diseases such as autosomal recessive intellectual disability (ARID) (Hennekam and Biesecker, 2012). This report highlights the detailed phenotypic evaluation following a multidisciplinary approach as the key for the identification of *SNX14* gene involvement.

## Conclusion

This study discloses the first non-consanguineous SCAR20 family caused by compound heterozygous pathogenic variants in *SNX14* gene. Additionally, this study also emphasizes laboratory-clinician crosstalk as key element to guide successful genetic investigation and, therefore, to increase ARID diagnostic yield. At the end, we broaden the *SNX14* phenotypic and mutational spectrum, describing two patients with dystonia and stereotypies beyond the typical clinical features of SCAR20, carrying p.(Arg399^*^) and a novel rearrangement encompassing introns 7 to 13, undetected by ES.

## Data Availability Statement

The original contributions presented in the study are included in the article, further inquiries can be directed to the corresponding author.

## Ethics Statement

The authors declare that the research was conducted in the absence of any commercial or financial relationships that could be construed as a potential conflict of interest. The study involving human participants were reviewed and approved by the medical ethical committee of the Centro Hospitalar Universit rio do Porto (CHUP, E.P.E.) – REF 2015.196 (168-DEFI/157-CES) – and ICBAS, UP – PROJETO N° 129/2015. Patient’s legal representatives signed informed consent for the use of DNA samples in intellectual disability research and biobanking, and for the publication of patient’s photographs.

## Author Contributions

NM: conception and study design, ES data analysis and interpretation, involved in drafting and writing. GS: collected the clinical data, co-writing medical results, interpretation and discussion. CS: shared molecular data analysis and interpretation. IM: data analysis and interpretation, involved in critical reviewing of the manuscript. BR: shared molecular data analysis and interpretation. RS and MP: critical revision, final review, and editing. AB: ES data interpretation, manuscript final critical revision and editing. TT: collected the clinical data, neurological history, co-writing medical results, interpretation and discussion, final review and editing. PJ: conception and study design, involved in drafting and manuscript final revision and editing. All authors gave approval to the final version of the manuscript.

## Conflict of Interest

The authors declare that the research was conducted in the absence of any commercial or financial relationships that could be construed as a potential conflict of interest.

## Footnotes

1 http://genome.uscs.edu/

2 http://conifer.sourceforge.net/

3 http://cadd.gs.washington.edu/score

4 http://hgvs.org/mutnomen/

5 http://www.noncode.org/index.php
